# Supplementary material for: Correlation between leukocyte phenotypes and prognosis of amyotrophic lateral sclerosis
Source: eLife. 2022 Mar 15;11:e74065. doi: 10.7554/eLife.74065 (PMC8923665; doi:10.7554/eLife.74065)
Supplement: Supplementary file 1. [file elife-74065-supp1.docx]

| Supplementary Table 1 Characteristics of the 92 patients with amyotrophic lateral sclerosis (ALS) included in the analysis of FlowC test, compared with the entire population of ALS patients during the study period in Stockholm, Sweden | | | |
| --- | --- | --- | --- |
| Characteristics | **Patients included in the analysis (N=92)** | **All patients in Stockholm (N=420)** | **P value for difference*** |
| Sex, N (%) |  |  | P=0.29 |
| Female | 49 (53%) | 201(48%) |  |
| Male | 43 (47%) | 219 (52%) |  |
| Age at diagnosis, years | |  | P=0.002 |
| Median (Q1,Q3) | 62 (54, 70) | 66 (57,72) |  |
| Diagnostic delay, months | |  | P=0.59 |
| Median (Q1,Q3) | 11.98 (7.52,19.89) | 12.35 (7.59,20.54) |  |
| Gene mutation, N (%)^+^ | |  | P=0.55 |
| *SOD1* | 3 (4.11%) | 9 (2.56%) |  |
| *C9orf72* | 10 (13.70%) | 30 (8.55%) |  |
| Other | 3 (4.11%) | 5 (1.42%) |  |
| Site of onset, N (%) | |  | P=0.03 |
| Limb | 64 (70%) | 250 (60%) |  |
| Bulbar | 20 (22%) | 118 (28%) |  |
| Other | 3 (3%) | 32 (8%) |  |
| Missing | 5 (5%) | 20 (5%) |  |
| Family history, N (%) | |  | P=0.60 |
| Yes | 7 (8%) | 30 (7%) |  |
| No | 35 (38%) | 201 (48%) |  |
| Not clear | 2 (2%) | 7 (2%) |  |
| Missing | 48 (52%) | 182 (43%) |  |
| N. of measurements for FlowC test (%) | |  |  |
| One | 10 (11%) | - |  |
| Two | 23 (25%) | - |  |
| Three | 23 (25%) | - |  |
| Four | 24 (26%) | - |  |
| Five | 10 (11%) | - |  |
| Six | 2 (2%) | - |  |
| *P value for the differences between patients included in the analysis and patients not included in the analysis; Wilcoxon rank sum test was used for the comparison of continuous variables whereas Chi-square test was used for the comparison of categorical variables.  ^+^Results available for 73 of the 92 patients included in the analysis, and 351 of the entire 420 patients in Stockholm. | | | |
