# Supplementary material for: Correlation between leukocyte phenotypes and prognosis of amyotrophic lateral sclerosis
Source: eLife. 2022 Mar 15;11:e74065. doi: 10.7554/eLife.74065 (PMC8923665; doi:10.7554/eLife.74065)
Supplement: Supplementary file 2. [file elife-74065-supp2.docx]

| **Supplementary Table 2** Mean levels of leukocyte subpopulations (N=288 patients) and lymphocyte subpopulations (N=92 patients) across all measures | | |
| --- | --- | --- |
| **Cell type** | **Mean (SD)** | **Normal range** |
| Leukocyte (10^9/L) | 7.8 (3.72) | 3.50-8.80 |
| Neutrophil (10^9/L) | 5.31 (3.01) | 1.60-5.90 |
| Lymphocyte (10^9/L) | 1.58 (0.6) | 1.10-3.50 |
| Monocyte (10^9/L) | 0.58 (0.22) | 0.20-0.80 |
| T cell (10^9/L) | 1.06 (0.37) | 0.65-1.57 |
| B cell (10^9/L) | 0.2 (0.17) | 0.08-0.28 |
| NK cell (10^9/L) | 0.21 (0.1) | 0.10-0.35 |
| CD4+ T cell (%) | 65.32 (11.2) | 44-79 |
| CD4+ naïve T cell (%) | 33.19 (14.54) | 22-62 |
| CD4+ EM (%) | 17.45 (10.76) | 10-47 |
| CD4+ CM (%) | 45.58 (13.02) | 14-48 |
| CD4+ EMRA (%) | 4 (7.39) | 1-9 |
| Th1 of CD4+ EM (%) | 49.19 (13.95) | 22-52 |
| Th2 of CD4+ EM (%) | 15.31 (10.67) | 4-27 |
| Th17 of CD4+ EM (%) | 12.4 (6.58) | 8-28 |
| Th1 of CD4+ CM (%) | 26.53 (5.46) | 13-30 |
| Th2 of CD4+ CM (%) | 26.87 (7.56) | 14-53 |
| Th17 of CD4+ CM (%) | 23.72 (4.91) | 20-37 |
| CD8+ T cell (%) | 30.2 (10.67) | 17-47 |
| CD8+ naïve T cell (%) | 22.27 (14.54) | 10-65 |
| CD8+ EM (%) | 24.64 (11.63) | 4-30 |
| CD8+ CM (%) | **15.81 (9.98)** | 1-12 |
| CD8+ EMRA (%) | 37.37 (18.41) | 18-68 |
| CD4+ HLA-DR+ CD38- (%) | 4.11 (2.85) | 2-11 |
| CD4+ HLA-DR+ CD38+ (%) | **3.24 (1.87)** | ≤2 |
| CD8+ HLA-DR+ CD38- (%) | 6.45 (5.03) | 2-22 |
| CD8+ HLA-DR+ CD38+ (%) | **8.84 (6.61)** | 1-5 |
| SD: standard deviation  Bold values indicate deviants from normal range. | | |
