# Supplementary material for: Correlation between leukocyte phenotypes and prognosis of amyotrophic lateral sclerosis
Source: eLife. 2022 Mar 15;11:e74065. doi: 10.7554/eLife.74065 (PMC8923665; doi:10.7554/eLife.74065)
Supplement: Supplementary file 3. [file elife-74065-supp3.docx]

| **Supplementary Table 3** Temporal changes of lymphocyte populations after diagnosis of amyotrophic lateral sclerosis (ALS), analysis of 92 patients with FlowC test* | | | | | | |
| --- | --- | --- | --- | --- | --- | --- |
| **Cell type** | **Unadjusted** | | | **Adjusted^+^** | | |
|  | **Coefficient** | **P value** | **FDR** | **Coefficient** | **P value** | **FDR** |
| T cell (10^9/L) | 0.02 | 0.26 | 0.58 | 0.01 | 0.63 | 0.81 |
| B cell (10^9/L) | 0.01 | 0.22 | 0.56 | 0.01 | 0.36 | 0.55 |
| NK cell (10^9/L) | -5.4E-04 | 0.91 | 0.92 | 4.1E-04 | 0.94 | 0.98 |
| CD4+ T cell (%) | 0.89 | 0.03 | 0.19 | 1.07 | 0.01 | 0.06 |
| CD4+ naïve T cell (%) | -0.93 | 0.03 | 0.19 | -1.16 | **0.01** | **0.04** |
| CD4+ EM (%) | -0.06 | 0.88 | 0.92 | 0.01 | 0.99 | 0.99 |
| CD4+ CM (%) | -0.25 | 0.56 | 0.75 | -0.09 | 0.83 | 0.96 |
| CD4+ EMRA (%) | 0.73 | 0.01 | 0.12 | 0.88 | **1.6E-03** | **0.01** |
| Th1 of CD4+ EM (%) | 0.65 | 0.31 | 0.58 | 0.69 | 0.31 | 0.51 |
| Th2 of CD4+ EM (%) | -0.52 | 0.32 | 0.58 | -0.67 | 0.24 | 0.50 |
| Th17 of CD4+ EM (%) | -0.14 | 0.60 | 0.76 | -0.02 | 0.94 | 0.98 |
| Th1 of CD4+ CM (%) | -0.17 | 0.47 | 0.68 | -0.28 | 0.26 | 0.50 |
| Th2 of CD4+ CM (%) | 0.48 | 0.11 | 0.36 | 0.66 | 0.03 | 0.13 |
| Th17 of CD4+ CM (%) | -0.32 | 0.12 | 0.36 | -0.27 | 0.20 | 0.50 |
| CD8+ T cell (%) | -0.37 | 0.33 | 0.58 | -0.50 | 0.20 | 0.50 |
| CD8+ naïve T cell (%) | 0.05 | 0.92 | 0.92 | -0.48 | 0.28 | 0.50 |
| CD8+ EM (%) | -0.64 | 0.13 | 0.36 | -0.29 | 0.51 | 0.73 |
| CD8+ CM (%) | 0.05 | 0.89 | 0.92 | 0.19 | 0.60 | 0.81 |
| CD8+ EMRA (%) | 0.43 | 0.45 | 0.68 | 0.70 | 0.23 | 0.50 |
| CD4+ HLA-DR+ CD38- (%) | 0.21 | 0.07 | 0.31 | 0.35 | **1.7E-03** | **0.01** |
| CD4+ HLA-DR+ CD38+ (%) | -0.06 | 0.42 | 0.68 | 0.02 | 0.84 | 0.96 |
| CD8+ HLA-DR+ CD38- (%) | 0.57 | 0.01 | 0.12 | 0.84 | **2.8E-04** | **0.01** |
| CD8+ HLA-DR+ CD38+ (%) | -0.07 | 0.81 | 0.92 | 0.37 | 0.25 | 0.50 |
| *Linear mixed model was applied to derive the coefficient estimates, per year and p value for trend.  ^+^Adjusted for age at diagnosis and sex.  FDR: False discovery rate. | | | | | | |
