# Supplementary material for: Correlation between leukocyte phenotypes and prognosis of amyotrophic lateral sclerosis
Source: eLife. 2022 Mar 15;11:e74065. doi: 10.7554/eLife.74065 (PMC8923665; doi:10.7554/eLife.74065)
Supplement: Supplementary file 4. [file elife-74065-supp4.docx]

**Supplementary Table 4** Associations of leukocyte populations with the risk of death after a diagnosis of amyotrophic lateral sclerosis (ALS), a cohort study of 288 patients with ALS in Stockholm, Sweden

| Cell type | HR (95%CI)* | P value | FDR |
| --- | --- | --- | --- |
| Leukocyte (10^9/L) | 1.16 (0.93-1.45) | 0.18 | 0.36 |
| Neutrophil (10^9/L) | 1.16 (0.94-1.44) | 0.17 | 0.36 |
| Lymphocyte (10^9/L) | 0.98 (0.78-1.23) | 0.85 | 0.85 |
| Monocyte (10^9/L) | 1.07 (0.86-1.32) | 0.55 | 0.73 |
| *Cox model was applied to derive the hazard ratios (HRs) with 95% confidence intervals (CIs) of risk of death, per standard deviation increase of the cell markers, with adjustment for age at diagnosis, sex, site of onset, diagnostic delay, ALSFRS-R score, time difference between the measure of ALSFRS-R score and diagnosis, BMI, and time difference between the measure of BMI and diagnosis.  FDR: false discovery rate. | | | |
