# Supplementary material for: Correlation between leukocyte phenotypes and prognosis of amyotrophic lateral sclerosis
Source: eLife. 2022 Mar 15;11:e74065. doi: 10.7554/eLife.74065 (PMC8923665; doi:10.7554/eLife.74065)
Supplement: Supplementary file 5. [file elife-74065-supp5.docx]

**Supplementary Table 5** Sensitivity analyses of the associations of leukocyte populations with risk of death after a diagnosis of amyotrophic lateral sclerosis (ALS), focusing on newly diagnosed ALS patients, first cell measure only, or excluding patients with *C9orf72* expansions*

| Cell type | HR (95%CI)^1^ | HR (95%CI)^2^ | HR (95%CI)^3^ |
| --- | --- | --- | --- |
| Leukocyte (10^9/L) | 1.13 (0.90-1.42) | 1.14(0.91-1.44) | 1.19 (0.95-1.51) |
| Neutrophil (10^9/L) | 1.15 (0.93-1.41) | 1.11(0.89-1.39) | 1.17 (0.94-1.46) |
| Lymphocyte (10^9/L) | 0.90 (0.74-1.09) | 1.02(0.82-1.27) | 1.02 (0.81-1.29) |
| Monocyte (10^9/L) | 0.97 (0.79-1.19) | 1.10(0.90-1.35) | 1.09 (0.88-1.36) |
| *Cox model was applied to derive the hazard ratios (HRs) with 95% confidence intervals (CIs) of risk of death, per standard deviation increase of the cell markers, with adjustment for age at diagnosis, sex, site of onset, diagnostic delay, ALSFRS-R score, time difference between the measure of ALSFRS-R score and diagnosis, BMI, and time difference between the measure of BMI and diagnosis.  ^1^Analysis restricted to newly diagnosed ALS patients.  ^2^Analysis restricted to the first measurement of cell populations.  ^3^Analysis restricted to patients without *C9orf72* mutation. | | | |
