# Supplementary material for: Correlation between leukocyte phenotypes and prognosis of amyotrophic lateral sclerosis
Source: eLife. 2022 Mar 15;11:e74065. doi: 10.7554/eLife.74065 (PMC8923665; doi:10.7554/eLife.74065)
Supplement: Supplementary file 6. [file elife-74065-supp6.docx]

| **Supplementary Table 6** Cross-sectional correlations between lymphocyte populations and ALS functional rating scale-revised (ALSFRS-R) score and disease progression rate, a cohort study of 92 ALS patients in Stockholm, Sweden* | | | | | | |
| --- | --- | --- | --- | --- | --- | --- |
| Cell type | ALSFRS-R | | | Progression rate | | |
|  | Coefficient | P value | FDR | Coefficient | P value | FDR |
| T cell(10^9/L) | 0.11 | 0.90 | 0.97 | -0.11 | 0.06 | 0.43 |
| B cell(10^9/L) | -0.85 | 0.31 | 0.95 | -0.05 | 0.20 | 0.50 |
| NK cell(10^9/L) | 0.57 | 0.51 | 0.95 | -0.10 | **0.02** | 0.43 |
| CD4+ T cell(%) | -0.06 | 0.96 | 0.97 | -0.04 | 0.32 | 0.62 |
| CD4+ naïve T cell(%) | 0.60 | 0.57 | 0.95 | -0.06 | 0.32 | 0.62 |
| CD4+ EM(%) | 1.17 | 0.22 | 0.95 | 0.06 | 0.22 | 0.51 |
| CD4+ CM(%) | -0.26 | 0.83 | 0.97 | 0.09 | 0.10 | 0.43 |
| CD4+ EMRA(%) | -0.71 | 0.53 | 0.95 | -0.10 | 0.09 | 0.43 |
| Th1 of CD4+ EM(%) | 0.09 | 0.93 | 0.97 | 0.02 | 0.80 | 0.88 |
| Th2 of CD4+ EM(%) | 0.86 | 0.31 | 0.95 | -0.10 | 0.11 | 0.43 |
| Th17 of CD4+ EM(%) | 0.35 | 0.75 | 0.97 | 0.03 | 0.65 | 0.84 |
| Th1 of CD4+ CM(%) | 1.40 | 0.18 | 0.95 | -0.01 | 0.87 | 0.91 |
| Th2 of CD4+ CM(%) | 0.03 | 0.97 | 0.97 | -0.09 | 0.16 | 0.46 |
| Th17 of CD4+ CM(%) | -0.58 | 0.58 | 0.95 | 0.04 | 0.53 | 0.83 |
| CD8+ T cell(%) | 0.40 | 0.73 | 0.97 | 0.07 | 0.15 | 0.46 |
| CD8+ naïve T cell(%) | 0.45 | 0.69 | 0.97 | -0.05 | 0.46 | 0.81 |
| CD8+ EM(%) | -1.06 | 0.37 | 0.95 | 0.02 | 0.79 | 0.88 |
| CD8+ CM(%) | 0.25 | 0.82 | 0.97 | 0.10 | 0.06 | 0.43 |
| CD8+ EMRA(%) | 0.66 | 0.55 | 0.95 | -0.04 | 0.54 | 0.83 |
| CD4+ HLA-DR+ CD38-(%) | -1.75 | 0.07 | 0.95 | -2.6E-03 | 0.97 | 0.97 |
| CD4+ HLA-DR+ CD38+(%) | -1.01 | 0.22 | 0.95 | -0.03 | 0.66 | 0.84 |
| CD8+ HLA-DR+ CD38-(%) | -0.65 | 0.57 | 0.95 | 0.03 | 0.59 | 0.84 |
| CD8+ HLA-DR+ CD38+(%) | -1.43 | 0.10 | 0.95 | -0.02 | 0.78 | 0.88 |
| *Generalized estimating equation model was applied to derive the coefficient estimates and p values, with adjustment for age at diagnosis and sex. ALSFRS-R score ranges from 0 to 48, with higher score showing better motor function status. Progression rate indicates the decline of motor function per month.  FDR: false discovery rate. | | | | | | |
