# Supplementary material for: Correlation between leukocyte phenotypes and prognosis of amyotrophic lateral sclerosis
Source: eLife. 2022 Mar 15;11:e74065. doi: 10.7554/eLife.74065 (PMC8923665; doi:10.7554/eLife.74065)
Supplement: Supplementary file 7. [file elife-74065-supp7.docx]

| **Supplementary Table 7** Sensitivity analyses of the associations of leukocyte populations with ALS functional rating scale-revised (ALSFRS-R) score and disease progression rate, after removing the blood samples with potential ongoing infection* | | | | | | |
| --- | --- | --- | --- | --- | --- | --- |
| Cell type | ALSFRS-R | | | Progression rate | | |
|  | Coefficient | P value | FDR | Coefficient | P value | FDR |
| Leukocyte (10^9/L) | -1.40 | 0.13 | 0.15 | 0.04 | 0.41 | 0.55 |
| Neutrophil (10^9/L) | -1.72 | 0.05 | 0.10 | 0.07 | 0.12 | 0.48 |
| Lymphocyte (10^9/L) | 1.28 | 0.15 | 0.15 | -0.07 | 0.41 | 0.55 |
| Monocyte (10^9/L) | -1.94 | **4.0E-03** | **0.02** | -0.01 | 0.90 | 0.90 |
| *Generalized estimating equation model was applied to derive the coefficient estimates and p values, with adjustment for age at diagnosis and sex.  ALSFRS-R score ranges from 0 to 48, with higher score showing better motor function status. Progression rate indicates the decline of motor function per month.  FDR: false discovery rate. | | | | | | |
