# Supplementary material for: Correlation between leukocyte phenotypes and prognosis of amyotrophic lateral sclerosis
Source: eLife. 2022 Mar 15;11:e74065. doi: 10.7554/eLife.74065 (PMC8923665; doi:10.7554/eLife.74065)
Supplement: Supplementary file 8. [file elife-74065-supp8.docx]

**Supplementary Table 8** Sensitivity analyses of associations of leukocyte populations with the risk of death after a diagnosis of amyotrophic lateral sclerosis (ALS), after removing the blood samples with potential ongoing infection*

| Cell type | HR (95%CI)* | P value | FDR |
| --- | --- | --- | --- |
| Leukocyte (10^9/L) | 1.06(0.84-1.34) | 0.62 | 0.79 |
| Neutrophil (10^9/L) | 1.07(0.85-1.34) | 0.58 | 0.79 |
| Lymphocyte (10^9/L) | 1.04(0.82-1.31) | 0.76 | 0.79 |
| Monocyte (10^9/L) | 0.97(0.76-1.23) | 0.79 | 0.79 |
| *Cox model was applied to derive the hazard ratios (HRs) with 95% confidence intervals (CIs) of risk of death, per standard deviation increase of the cell markers, with adjustment for age at diagnosis, sex, site of onset, diagnostic delay, ALSFRS-R score, time difference between the measure of ALSFRS-R score and diagnosis, BMI, and time difference between the measure of BMI and diagnosis.  FDR: false discovery rate. | | | |
